# Editorial: Psychological Aspects of Cannabis Use and Cannabis Use Disorder

**DOI:** 10.3389/fpsyt.2021.789197

**Published:** 2021-11-05

**Authors:** Daniel Feingold, Eva Hoch, Aviv Weinstein, Wayne Hall

**Affiliations:** ^1^Department of Psychology, Ariel University, Ariel, Israel; ^2^Clinic and Policlinic of Psychiatry and Psychotherapy, Clinic of Ludwig-Maximilian-University, Munich, Germany; ^3^Division of Clinical Psychology and Psychological Treatment, Department of Psychology, Ludwig-Maximilian-University, Munich, Germany; ^4^National Centre for Youth Substance Use Research, The University of Queensland, St Lucia, QLD, Australia

**Keywords:** cannabis, marijuana, cannabis use disorder, psychology, cognition, emotion

An increasing global prevalence of cannabis use has produced increased treatment seeking for Cannabis Use Disorder (CUD) and an increased research effort to identify factors associated with initiation of cannabis use, transitions to regular cannabis use and the onset of CUD (1, 2). The majority of empirical studies focus on biological and psychiatric aspects of cannabis use and CUD, including the role of genetic and neurological factors as well as comorbid mental disorders in studying the etiology and phenomenology of cannabis use and CUD (3, 4). In recent years, emerging evidence points to the contribution of psychological, cognitive, and motivational factors to cannabis use and CUD (5).

In this Research Topic, we assembled a collection of research focusing on psychological aspects of cannabis use and CUD that brought together researchers from various psychological schools who employed diverse methodological practices (e.g., experimental research, narrative studies, theoretical writing). This collection of papers reviews the evidence which has been accumulated in that field, presents up-to-date findings, describes gaps in our knowledge and identifies future directions in research, practice, and policy.

Sorkhou et al. systematically reviewed 124 cross-sectional and longitudinal studies from 1990 to 2020 on adverse behavioral outcomes in cannabis users who did not have psychiatric and medical co-morbidities. The preponderance of the evidence suggested that the risks of adverse outcomes increased with the frequency of cannabis use, the THC (but not CBD) content of cannabis used, age of onset, and cumulative cannabis exposure. The strongest evidence was for psychosis and psychosocial functioning.

Preuss et al. reviewed systematic reviews, meta-analyses, and relevant papers published within the last decade on the contribution of cannabis use to car crashes. Meta-analyses and culpability studies consistently found a modest but significantly increased risk of crashes after acute cannabis use. These risks varied by study type, crash severity, and the method used to measure cannabis use. Some studies show a significant correlation between high THC blood concentrations and car crash risk but most studies did not find a relationship at lower THC concentrations. They did not find any scientifically supported cut-off concentration of THC in blood that could be used to define impaired driving. Further research was needed to assess dose-response effects of cannabis use on neuropsychological functioning related to driving skills and crash risk.

Brands et al. discuss key questions regarding the possible effect of cannabis legalization on impaired driving and road safety. According to the authors, emerging evidence indicate that driving under the influence of cannabis may increase the risk for collision and contribute to deaths and injuries resulting from collisions and young adults are the most likely to drive under the influence of cannabis. The acute effects of cannabis on driving-related behaviors include an increase in weaving, reduction in speed, and prolonged reaction time. The authors call for further research, exploring topics such as the specific effects of cannabis use on collisions types and injury severity, sex differences in the effects of cannabis and the impairing effects of medical cannabis use on driving.

López-Pelayo et al. used Classification and Regression Trees (CART) analyses to identify the independent and integrated effects of cannabis use patterns on self-reported cannabis-related harms in a large sample of cannabis users. The results indicated that early onset of regular cannabis use and current frequent cannabis use increased the probability of risky alcohol use. In addition, early onset of regular cannabis use in combination with prolonged regular use was associated with increased odds of a motor vehicle accident. Current daily or near daily cannabis use was independently associated with screening positive for a cannabis use disorder.

Leung et al. reviewed studies conducted between 1973 and 2020 that examined whether cannabis users who used higher THC content cannabis products can and do titrate the doses of THC that they receive. They included (1) experimental laboratory studies of dose titration; (2) observational studies of users of more potent products; and (3) surveys on whether cannabis users titrate when using more potent products. Some experiments found inverse associations between the THC content of cannabis and the amount smoked and smoking topography but in others higher THC doses were consumed and more marked psychological and physiological effects observed. In some surveys, cannabis users reported that they use less of more potent cannabis products, but in other surveys, persons who used more potent cannabis reported more adverse effects of use, suggesting that they received higher THC doses. They concluded that we need better experimental and epidemiological research to inform regulatory policies to minimize harms from the use of high THC cannabis products.

De la Peña-Arteaga et al. systematically reviewed studies of the association between exposure to childhood physical and sexual abuse and adolescent cannabis use. They included 13 studies, eight of which had a low risk of bias. Eleven papers found a modest relationship between childhood sexual abuse and adolescent cannabis use [OR 1.29 (95% CI 1.08–1.49)] and 7 found a modest relationship between childhood physical abuse and adolescent cannabis use [OR 1.39 (95% CI 1.12–1.66]. The strength of the evidence varied with the method of exposure ascertainment. There was some evidence of differences in association by gender, age of cannabis initiation, and the severity of the abuse. Further work is needed on the role played by adolescent cannabis use in the causal pathway between childhood abuse and adult mental health problems.

Claus et al. assess the relationship between the severity of cannabis withdrawal syndrome (CWS) and urine cannabinoid concentrations in 78 adult cannabis-dependent subjects. They used a commercial enzyme immunoassay of 11-nor-9-carboxy-Delta-9-tetrahydrocannabinol (THC-COOH) to assess subjects 13 times during a 24-day inpatient detoxification treatment. Absolute urinary THC-COOH levels were significantly correlated with Marijuana Withdrawal Checklist scores (*r* = 0.248; *p* < 0.001) but after adjustment for serial creatinine ratios the correlation was significant only in the sample with higher MWC scores (>11 points) at admission (*n* = 21; *r* = 0.247; *p* = 0.002). These relationships persisted when they examined day-to-day change in THC-COOH-levels. MWC scores were significantly correlated with the Clinical Global Impression-Severity (CGI-S; *r* = 0.812; *p* < 0.001). Females showed a significantly slower decline in urine THC-COOH levels and more prolonged CWS course and substantial illness severity (per CGI-S) in nearly 30% of cases.

Gullo et al. explored the utility of a bioSocial Cognitive Theory in treating cannabis use disorders. Social Cognitive Theory (SCT) emphasizes the importance of targeting two psychological mechanisms: drug outcome expectancies and low drug refusal self-efficacy. They outlined a new bioSocial Cognitive Theory (bSCT) that integrated findings from the literature and presented preliminary evidence that treatment based on this approach improved outcomes in persons with cannabis use disorders.

Serebro et al. undertook a narrative exploration of cannabis use disorder among young Israeli combat veterans who used cannabis to cope with PTSD symptoms. They used narrative analysis to interpret retrospective in-depth interviews with 12 combat veterans who were released from mandatory military duty during the past 5 years and qualified for a diagnosis of a CUD. Participants came from a larger quantitative study of veterans who screened positive for a diagnosis of CUD on the Cannabis Use Disorder Identification Test- Revised (CUDIT-R) questionnaire. Five main themes were identified: (a) traumatic events, (b) attitudes toward cannabis use, (c) combatant identity, (d) the role of authority/father figures, and (e) moral crisis. A meta-theme was “from enchantment to disillusion” which represented a gradual shift from a hopeful, highly motivated stance into a state of mental rupture and moral injury, which they unsuccessfully treated by their excessive use of cannabis. This study highlighted the role that use of cannabis for “self-medication” of trauma symptoms contributed to a sense of betrayal.

Lorenzetti et al. assessed the residual effects of chronic cannabis use and abstinence on verbal and visuospatial learning. Regular cannabis users differ from non-using controls in learning performance but it is unclear (i) if these differences are specific to distinct domains of learning (verbal, visuospatial), (ii) if these differences increase with cannabis exposure and (iii) if they dissipate after sustained abstinence. They examined different domains of learning (verbal, visuospatial) in current and abstaining cannabis users, and the role of chronicity of use in 127 psychiatrically healthy participants (65 female) with mean aged of 34 years, of whom 69 were current regular cannabis users (mean 15 years use), 12 were former cannabis users who had been abstinent for ~2.5 years (after 16 years use), and 46 were non-cannabis using controls. Current cannabis users performed worse than non-users on verbal learning (Long Delay Cued Recall) and visuospatial learning (Retroactive Interference and LD Rotated Recall). Prolonged abstinence was associated with altered verbal learning but intact visuospatial learning.

López-Pelayo et al. argue that a standardized measure of cannabis dose is a priority for research that will inform policy-making, the design of clinical and harm-reduction interventions and improve consumer safety. They propose that a Standard Joint Unit (SJU) be developed for cannabis. A back-casting foresight method was used to achieve consensus on developing an SJU with 32 professionals from 13 countries and 10 disciplines. Several characteristics of the SJU were defined: (1) core values: easy-to use, universal, focused on THC, accurate, and accessible; (2) key challenges: sudden changes in patterns of use, heterogeneity of cannabis products and administration routes, variations over time in THC concentrations and laws that regulate recreational and medical cannabis use; and (3) facilitators: previous experience with standardized measurements, funding opportunities, multi-stakeholder support, high prevalence of cannabis users, and widespread changes in legislation. Participants identified three steps for the implementation of a SJU by 2030: (1) building a task-force to develop a consensus-based SJU; (2) expanding national-level data; and (3) linking SJU consumption to “risky use” based on evidence of harms.

Sofis et al. conducted an exploratory study on the effect of cannabis use frequency and training on episodic memory, specifically on the recall of specific and rewarding events. Active cannabis users were randomly assigned to receive a brief intervention aimed at enhancing specificity of event retrieval (Episodic Specifity Induction: ESI) or a control group. They were categorized according to their intensity of past-month cannabis use. Results indicated higher levels of vividness and excitement ratings in the low vs. high intensity and ESI vs. control groups. No significant interaction was observed, suggesting that frequent cannabis use may be associated with the retrieval of less specific and rewarding events, which may be compensated by ESI.

Allick et al. conducted a systematic review and meta-analysis of voxel-based morphometry studies of cortical gray matter volume (GMV) in adolescent (12–21 years old) cannabis users. They used PRISMA guidelines and effect-size seed-based d mapping meta-analyses to compare age- and sex-related differences between cannabis using and typically developing youth. Six whole-brain voxel-based morphology studies were analyzed that included 357 cannabis users and 404 non-users. Meta-analysis did not identify any region showing significant GMV differences but age and sex differences were identified in meta-regressions: younger cannabis users showed increased superior temporal gyrus (STG) volume and older users showed decreased STG compared to age-matched controls. The authors conclude that GMV abnormalities in teen cannabis users are subtle and may be partially attributed to age and sex differences.

In conclusion, cannabis can have both therapeutic effects and adverse consequences [see (3, 6) for reviews]. This collection of papers has evaluated the behavioral and cognitive outcomes of cannabis including driving safety and verbal and visuospatial learning. Other studies looked at the association between exposure to childhood physical and sexual abuse and adolescent cannabis use and the use of cannabis for treating PTSD symptoms. The pharmacology of dose titration and the association between measures of THC and withdrawal has also been explored. Finally, studies have evaluated outcome of treatment and the residual effects of chronic cannabis use and abstinence on verbal and visuospatial learning in adult users and cortical gray matter volume in adolescent cannabis users. We hope that this collection will be a positive contribution to this exciting field of research.

## Author Contributions

DF, WH, and AW wrote sections of the manuscript. All authors contributed to manuscript revision, read, and approved the submitted version.

## Conflict of Interest

The authors declare that the research was conducted in the absence of any commercial or financial relationships that could be construed as a potential conflict of interest.

## Publisher's Note

All claims expressed in this article are solely those of the authors and do not necessarily represent those of their affiliated organizations, or those of the publisher, the editors and the reviewers. Any product that may be evaluated in this article, or claim that may be made by its manufacturer, is not guaranteed or endorsed by the publisher.
